# Genome-wide analysis of three histone marks and gene expression in *Paulownia fortunei* with phytoplasma infection

**DOI:** 10.1186/s12864-019-5609-1

**Published:** 2019-03-21

**Authors:** Lijun Yan, Guoqiang Fan, Xiaoyu Li

**Affiliations:** 1 Institute of Paulownia, Henan Agricultural University, Zhengzhou, Henan 450002 People’s Republic of China; 2 College of Forestry, Henan Agricultural University, Zhengzhou, Henan 450002 People’s Republic of China

**Keywords:** PaWB, Epigenetics, Histone modifications, ChIP-Seq

## Abstract

**Background:**

Paulownia withes’-broom (PaWB) disease caused by phytoplasma is a serious infectious disease for Paulownia. However, the underlying molecular pathogenesis is not fully understood. Recent studies have demonstrated that histone modifications could play a role in plant defense responses to pathogens. But there is still no available genome-wide histone modification data in non-model ligneous species infected with phytoplasma.

**Results:**

Here, we provided the first genome-wide profiles of three histone marks (H3K4me3, H3K36me3 and H3K9ac) in *Paulownia fortunei* under phytoplasma stress by using chromatin immunoprecipitation sequencing (ChIP-Seq). We found that H3K4me3, H3K36me3 and H3K9ac were mainly enriched in the genic regions in *P. fortunei* with (PFI) and without (PF) phytoplasma infection. ChIP-Seq analysis revealed 1738, 986, and 2577 genes were differentially modified by H3K4me3, H3K36me3 and H3K9ac marks in PFI under phytoplasma infection, respectively. The functional analysis of these genes suggested that most of them were mainly involved in metabolic pathways, biosynthesis of secondary metabolites, phenylpropanoid biosynthesis, plant-pathogen interaction and plant hormone signal transduction. In addition, the combinational analysis of ChIP-Seq and RNA-Seq showed that differential histone methylation and acetylation only affected a small subset of phytoplasma-responsive genes.

**Conclusions:**

Taken together, this is the first report of integrated analysis of histone modifications and gene expression involved in Paulownia-phytoplasma interaction. Our results will provide the valuable resources for the mechanism studies of gene regulation in non-model plants upon pathogens attack.

**Electronic supplementary material:**

The online version of this article (10.1186/s12864-019-5609-1) contains supplementary material, which is available to authorized users.

## Background

Plants are constantly subject to a wide variety of pathogens that threaten their growth and survival. As sessile organisms, plants are unlike animals since they cannot relocate to evade the adverse conditions. Besides this, they also lack somatic adaptive immune system that can produce antibodies and lack mobile defender cells to detect and prevent infection. Instead, they have evolved sophisticated and multilayered innate immune systems, including recognition, signal transduction, and defense responses, to counteract these threats [1]. The stimulation of plant defense response to pathogens involves in reprogramming of plant transcription upon recognition of pathogen infection, which is central for launching robust and effective host defense responses [2]. For instance, in phytoplasma-infected Paulownia, dramatic changes of gene expression profile have been revealed, and defense-related genes were significantly induced [3, 4]. Similarly, alterations in gene expression have also been reported in *Citrus aurantifolia* [5], *Vitis vinifera* [6, 7], *Cocos nucifera* [8, 9], *Catharanthus roseus* [10] after phytoplasma infection. Recent evidences have demonstrated that plants utilize the epigenetic control of gene expression to fine-tune their defense when challenged by pathogens [11]. Histone modifications are deemed to be one of the most important epigenetic regulation, and always occur at the amino-terminal tails of each histone. Histone modifications can change the higher-order structure of chromatin and orchestrate the DNA-based processes (such as transcription, repair, replication and recombination) by affecting the interaction of histones with DNA or by recruiting ordered enzyme complexes to chromatin [12–14]. Histone modifications are implicated in transcriptional regulation, and the effects of histone modifications on gene expression depend on the modification types and degree. In general, an open chromatin state increases the accessibility of the genome to the transcriptional apparatus, thereby activating gene transcription. Whereas a closed chromatin state is associated with transcriptional repression [15]. For instance, histone acetylation (e.g. H3K9ac) almost invariably correlates with transcriptional activation. By contrast, methylation of histone H3 lysine 4, lysine 36 and lysine 79 (H3K4, H3K36 and H3K79) are connected to active transcription, while methylation at H3K9, H3K27, and H4K20 sites are implicated in repressed transcription [14, 16]. It has been suggested that distinct histone modifications can act sequentially or in a combinatorial fashion to bring about distinct transcriptional outcomes [17], thus ultimately influencing plants differentiation, development, growth and their responses to biotic and abiotic stresses [18, 19]. Histone modifications are particularly important epigenetic regulation mechanisms involved in plants defense responses to pathogens infection, and histone acetylation/deacetylation and histone methylation/demethylation have clearly been shown to activate or repress position-dependent transcription of target genes [11, 20]. For instance, Ayyappan et al. [21] conducted genome-wide analysis of changes on histone modification (H3K9me2 and H4K12ac) and gene expression in *Phaseolus vulgaris* under rust pathogen (*Uromyces appendiculatus*) stress, and found that the methylation and acetylation patterns of *P. vulgaris* altered in response to rust pathogen, affecting a large proportion of defense-related genes expression, including plant resistant (R) genes, detoxifying enzymes and genes associated with ion flux and cell death. Additionally, some studies found the involvement of some histone modifying enzymes in plants response to pathogens infection. For example, Ding et al. [22] demonstrated that the expression level of HDT701, a member of the plant-specific HD2 subfamily of histone deacetylases (HDACs) in rice, altered after infection with fungal pathogen *Magnaporthe oryzae*. And it negatively regulates innate immunity of rice by modulating the levels of histone H4 acetylation of pattern recognition receptor (PRR) and defense-related genes. Choi et al. [23] reported that HDA19 directly targeted the promoters of pathogenesis related 1 (PR1) and PR2, and is involved in the repression of salicylic acid (SA)-mediated basal defense responses in Arabidopsis. Arabidopsis histone methyltransferase SET domain group 8 (SDG8) were found to be required for plant defense against necrotrophic fungal pathogens *Alternaria brassicicola* and *Botrytis cinerea* through H3K36me3-mediated activation of a subset of genes involved in the jasmonic acid/ethylene (JA/ET) signaling defense pathway [24].These evidences demonstrated that histone modifications are implicated in the defense-associated transcriptional reprogramming in plants upon pathogen challenge. However, information about histone marks in plants infected with phytoplasma is still lacking.

Paulownia is a deciduous tree species indigenous to China with great economic and ecological values because of its good traits, such as fast-growing, high biomass and high-quality wood, and it has been extensively used in afforestation, furniture making, solid biofuel and cellulose pulp [25, 26]. However, the growth and biomass of Paulownia is severely affected by Paulownia witches’ broom (PaWB) disease. PaWB disease is caused by phytoplasma, which is a serious infectious disease for Paulownia. Phytoplasmas are wall-less pleiomorphic phytopathogenic bacteria of the class Mollicutes with a single cell membrane and small genome size [27]. Because of their reduced genome, phytoplasmas lack most essential metabolic pathways [28–30]. Although phytoplasma has been tried to cultivate in vitro [31, 32], their culture in complex artificial media is still difficult. Phytoplasma is transmitted by primarily phloem-sucking insects such as leafhoppers (Cicadellidae, e.g. *Empoasca flavescens*), planthoppers (Fulgoromorpha, e.g. *Hyalesthes obsoletus*) and psyllids (Psyllidae, e.g. *Cacopsylla picta*), and can also be spread through the parasitic plant dodder (*Cuscuta* spp.) and vegetative propagation [33, 34]. In infected plants, phytoplasmas inhabit almost exclusively in the phloem sieve tube elements and spread systemically throughout the plant via moving through the pores of phloem sieve plates [35]. Paulownia infected with phytoplasma results in various symptoms, including witches’ broom, phyllody, yellowing, phloem necrosis, stunting and short internodes, leading to huge economic losses to Paulownia production [36]. However, the underlying molecular pathogenesis of PaWB disease remains poorly understood, because phytoplasmas are uncultivable in vitro [37]. Over the past few decades, many researchers have been engaged in the research of PaWB disease, and most of them focused on the characteristics of PaWB phytoplasma [38–42] and morphological, physiological, biochemical and molecular changes of Paulownia after phytoplasma infection [43–48]. Recently, many researchers began to explore how Paulownia responded to phytoplasma at transcriptional, post-transcriptional and translational levels, and numerous mRNAs, micro RNAs (miRNAs), metabolites, proteins and long noncoding RNAs (lncRNAs) putatively associated with PaWB disease have been reported [49–53]. At the epigenetic level, Cao et al. [54] investigated effect of phytoplasma infection on DNA methylation of Paulownia by using amplified fragment length polymorphism (AFLP) and methylation-sensitive amplification polymorphism (MSAP), and found that phytoplasma infection did not change the DNA sequence of Paulownia at the AFLP level, but altered the global DNA methylation levels and patterns. But until now, there have been no reports on the genome-wide profiles of histone modifications in Paulownia with phytoplasma infection, and their regulation roles in gene expression and Paulownia-phytoplasma interaction is still unknown at epigenome level.

Trimethylation of histone H3 lysine 4 (H3K4me3), trimethylation of histone H3 lysine 36 (H3K36me3) and acetylation of histone H3 lysine 9 (H3K9ac) are three well-studied histone marks. Previous studies have demonstrated that H3K4me3 and H3K9ac are enriched downstream of the transcription start sites (TSS) of genes, while H3K36me3 is prevalent in the gene body, and these histone marks are all associated with gene activation [55, 56]. Compared with previous research methods used in histone modification researches, such as chromatin immunoprecipitation followed by PCR (ChIP-PCR) or microarray hybridization (ChIP-chip), chromatin immunoprecipitation coupled with next-generation sequencing (ChIP-Seq) has proven to be an effective technology to perform systematic genome-wide studies of histone modifications with higher resolution and sensitivity, and it has been widely used in Arabidopsis [55, 57], rice [58, 59], cotton [60] and *Eucalyptus grandis* [61]. Herein, we report the epigenomic profiles of these three histone modifications in Paulownia by using ChIP-Seq, and investigate whether they are involved, to what extent, in the responses of Paulownia to phytoplasma. Furthermore, we also performed the integrated comparative analysis of the histone modification patterns and gene expression profiles to determine the association between them.

## Results

### The morphological changes of *P. fortunei* in response to phytoplasma

**Fig. 1:**
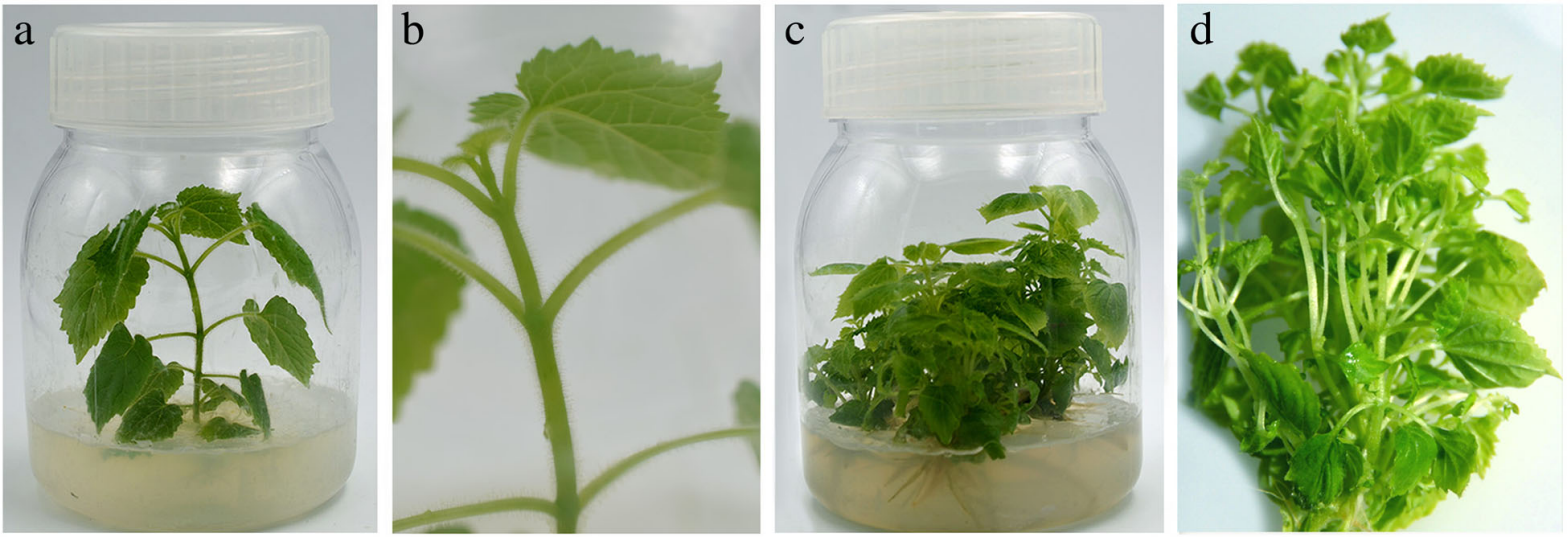
The morphological characteristics of *P. fortunei* (**a**, **b**) and *P. fortunei* with phytoplasma infection (**c**, **d**)

PFI displayed distinct changes in morphology compared with PF. As showed in Fig. 1, the leaves of PFI were smaller and thinner than those of PF. The plantlet of PF was robust with dense bristles on its leaves and stem, whereas PFI plantlet had no bristles. The leaf color of PFI plantlet was lighter than that of PF. The most obviously morphological difference between them was that PFI plantlet had numerous axillary buds, while PF plantlet had no axillary buds with it.

### Genome-wide patterns of histone modifications in Paulownia under phytoplasma stress

**Fig. 2:**
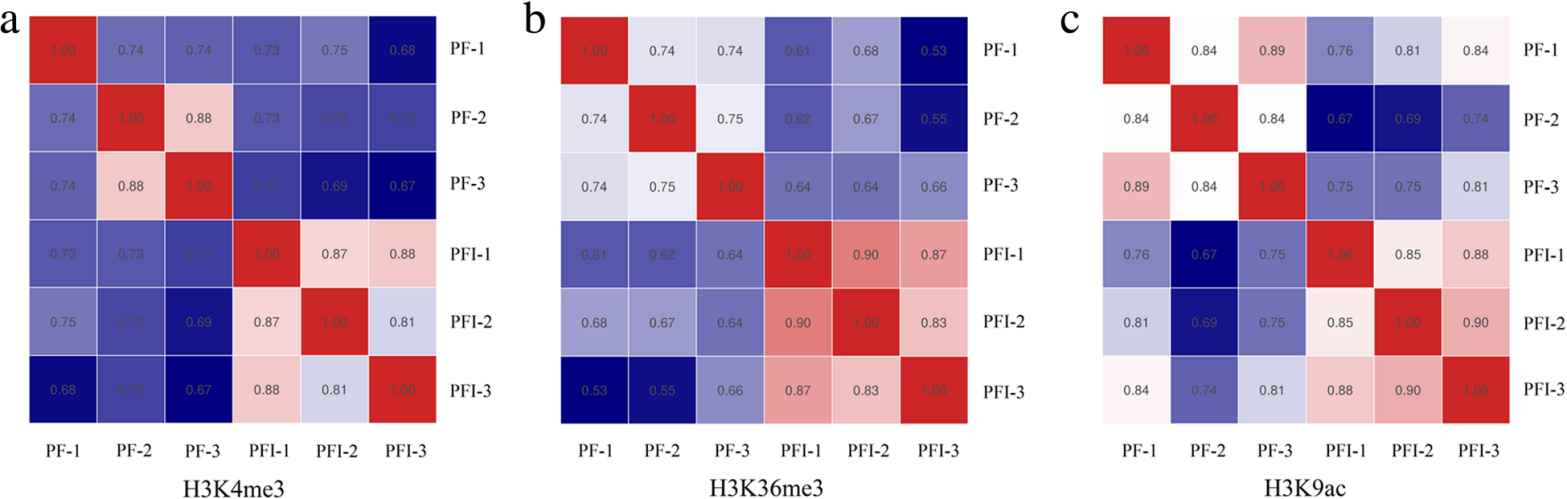
The correlation between each biological replicates of *P. fortunei* and *P. fortunei* with phytoplasma infection for H3K4me3 (**a**), H3K36me3 (**b**) and H3K9ac (**c**) marks

**Fig. 3:**
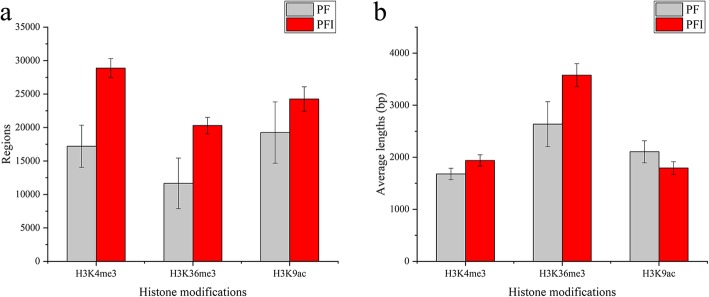
Genome-wide patterns of H3K4me3, H3K36me3 and H3K9ac in *P. fortunei* and *P. fortunei* with phytoplasma infection. **a** Numbers of histone modified regions detected by MACS software; **b** Average lengths of histone modified regions

**Fig. 4:**
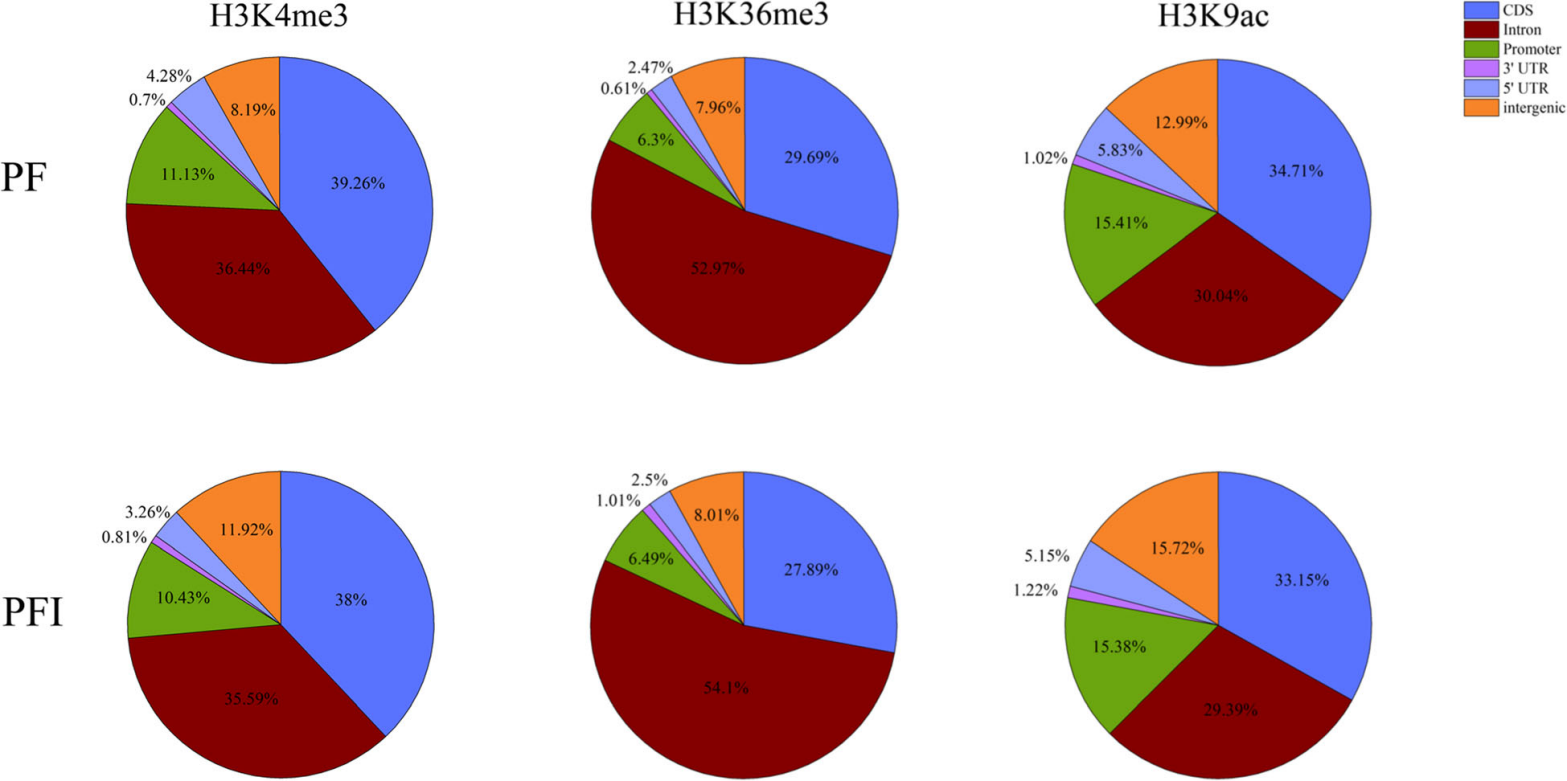
The distribution patterns of H3K4me3, H3K36me3 and H3K9ac within different regions in Paulownia genome

**Fig. 5:**
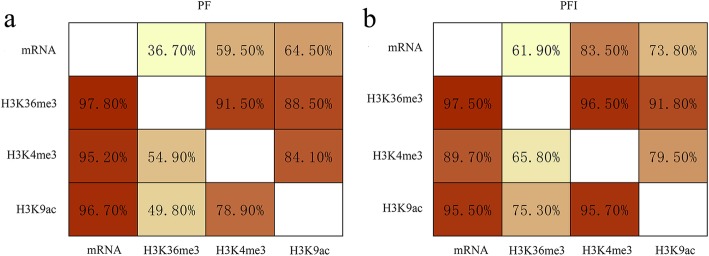
The concurrence frequencies for histone modifications (H3K4me3, H3K36me3, H3K9ac) and mRNA of *P. fortunei* (a) and *P. fortunei* with phytoplasma infection (b). The percentage number indicates the frequency that histone modification or mRNA on the x-axis co-occurs with a given histone modification or mRNA on the y-axis. mRNA represents the expressed gene

To investigate whether histone modifications (H3K4me3, H3K36me3 and H3K9ac) are implicated in the responses of Paulownia to phytoplasma, we applied ChIP-Seq to determine the changes of histone modifications between PF and PFI at the whole genome level. After preprocessing the raw reads, a total of 259, 271 and 276 million clean reads were derived from H3K4me3, H3K36me3 and H3K9ac histone modifications, respectively. Over 76% of the ChIP-Seq reads could be aligned to the *P. fortunei* reference genome. The detailed statistics of ChIP-Seq for H3K4me3, H3K36me3 and H3K9ac in Paulownia were summarized in Additional file 1: Table S1. The reproducibility between biological replicates is shown in Fig. 2, indicating that the results were reliable. To analyze the extent of histone modifications at a whole genome level, genomic regions associated with H3K4me3, H3K36me3 and H3K9ac were determined by MACS software. In total, 17,202 and 28,887 enriched regions for H3K4me3, 11,652 and 20,298 enriched regions for H3K36me3, and 19,255 and 24,256 enriched regions for H3K9ac were identified in PF and PFI, respectively. Among these histone marks, H3K4me3 and H3K9ac were found at high frequencies. We also found that the number of regions modified by these three histone marks were higher in PFI compared to PF. This result indicated that alterations of histone modifications occurred at genome-wide in PF after phytoplasma infection (Fig. 3a). The lengths of histone-modified regions varied dramatically in PF and PFI, and histone marks (Fig. 3b). As shown in Fig. 3, H3K36me3 was located in relatively few regions, but their average length was significantly longer than the other two histone marks. To investigate the distribution of peaks detected in the ChIP-Seq along the Paulownia genome, we classified the Paulownia genome into six classes of genomic regions, including promoter, 5′ UTR, 3′ UTR, CDS, intron and intergenic regions. It was found that these three histone marks were mainly biased towards the genic regions (promoter, 5′ UTR, 3′ UTR, CDS, intron) in both PF and PFI (84.29–92.04%) (Fig. 4). However, the ratios of the three histone modifications in each genomic region showed histone mark-specific patterns and differed between PF and PFI. For example, the proportions of H3K4me3 and H3K9ac peaks deposited in the CDS regions (33.15–39.26%) were almost equal to those deposited in the intron regions (29.39–36.44%), while H3K36me3 marks was more likely to be found within the intron regions but less likely to be found within the CDS regions compared with the other two histone modifications, and the ratios of H3K36me3 peaks deposited in the intron regions (52.97–54.11%) was approximately two times as much as those deposited in the CDS regions (27.89–29.69%). To analyze the relationships among the histone marks (H3K4me3, H3K36me3 and H3K9ac) and their correlations with gene activity, we examined the concurrence frequencies for these three histone modifications and gene expression. The results showed that the majority of genes in PF and PFI were co-marked by at least two histone modifications (Fig. 5). Furthermore, we noticed that a large proportion of genes modified by H3K36me3 were also co-modified by H3K4me3 (91.5% in PF; 96.5% in PFI) or H3K9ac (88.5% in PF; 91.8% in PFI), while a much smaller number of genes occupied by H3K4me3 (54.9% in PF; 65.8% in PFI) or H3K9ac (49.8% in PF; 75.3% in PFI) also contained H3K36me3. As expected, high concurrence frequencies were discovered between gene expression and the three histone modifications.

### Differentially histone modified genes in response to phytoplasma in Paulownia

**Fig. 6:**
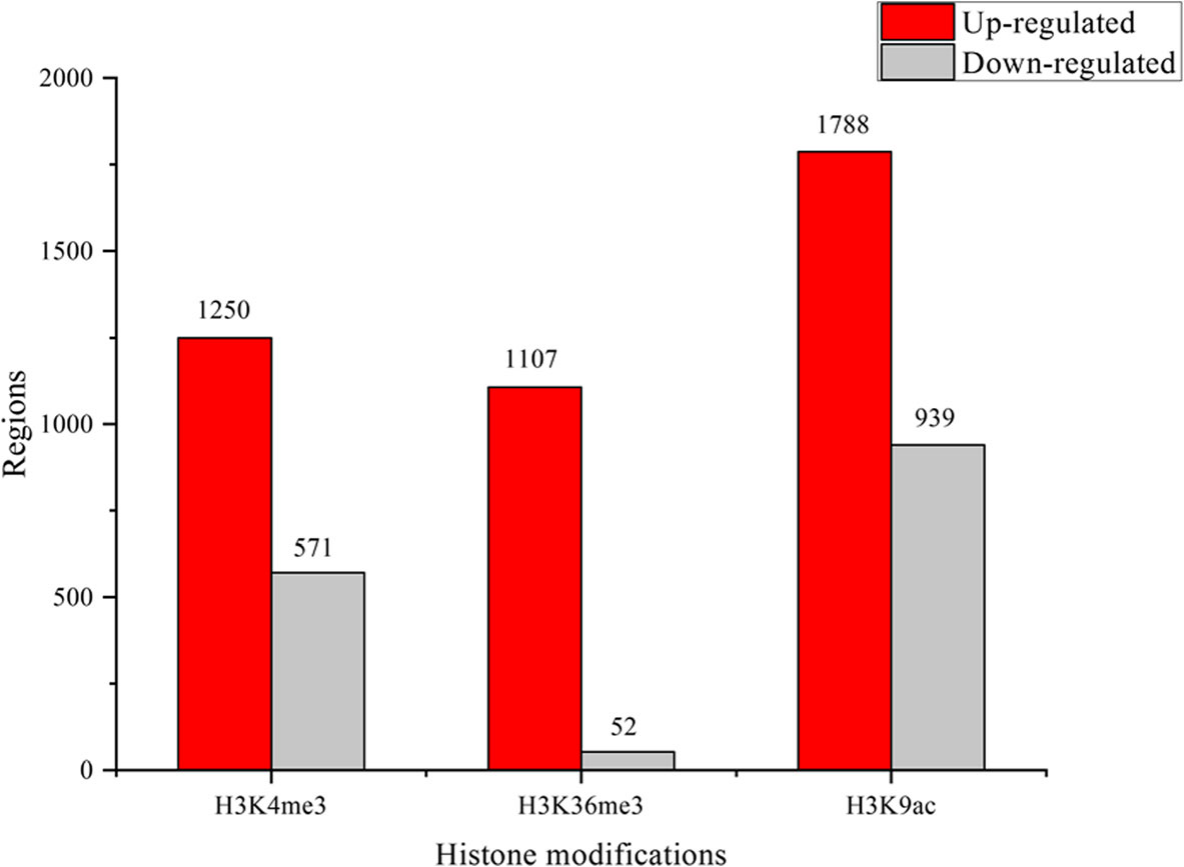
Numbers of differentially modified regions in *P. fortunei* with phytoplasma infection compared to *P. fortunei* for H3K4me3, H3K36me3 and H3K9ac marks

**Fig. 7:**
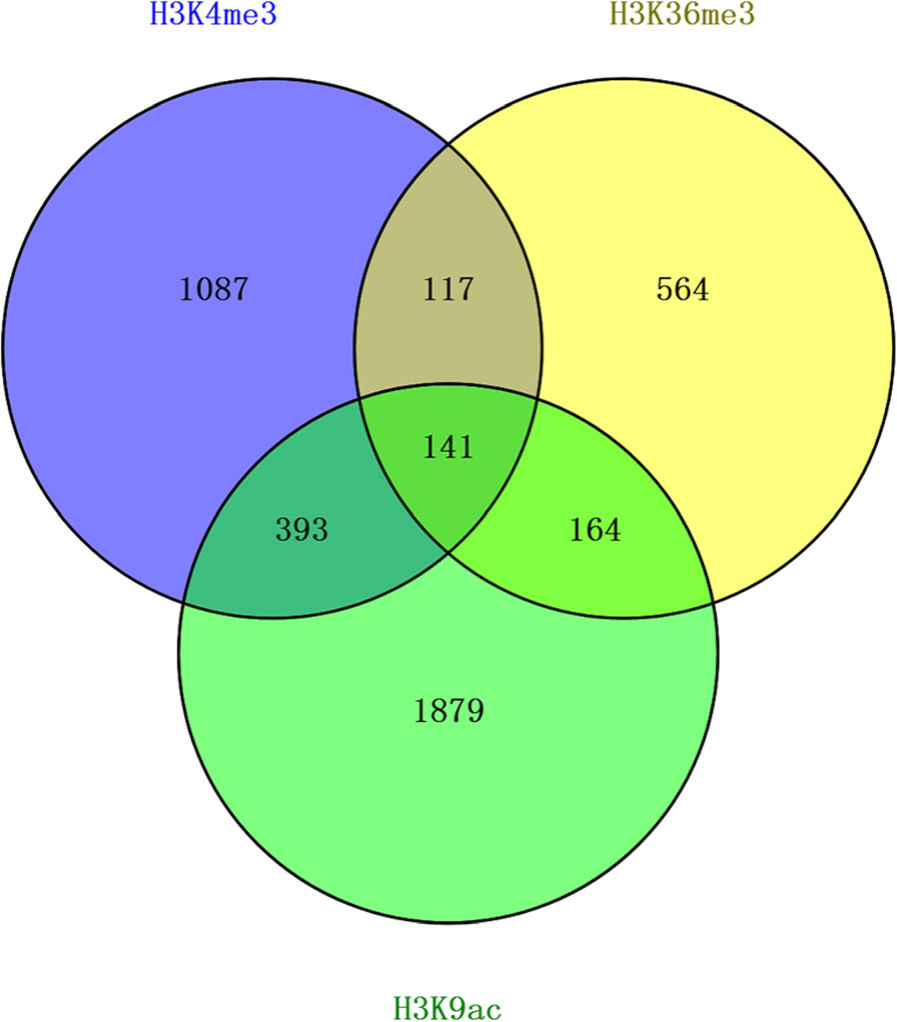
Venn diagram of differentially modified genes by H3K4me3, H3K36me3 and H3K9ac marks in *P. fortunei* with phytoplasma infection compared to *P. fortunei*

**Fig. 8:**
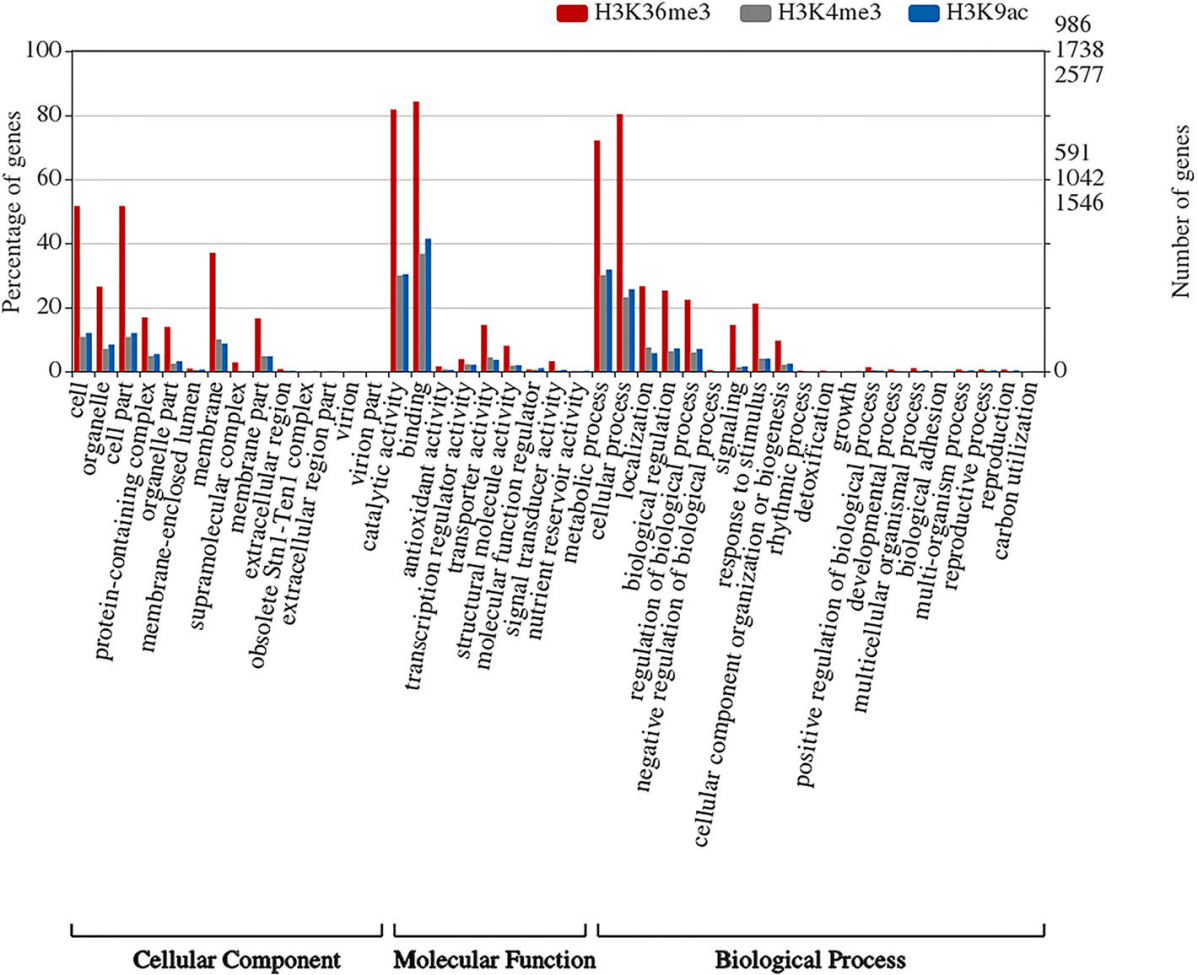
GO analysis of genes differentially modified by H3K4me3, H3K36me3 and H3K9ac marks under phytoplasma stress

**Fig. 9:**
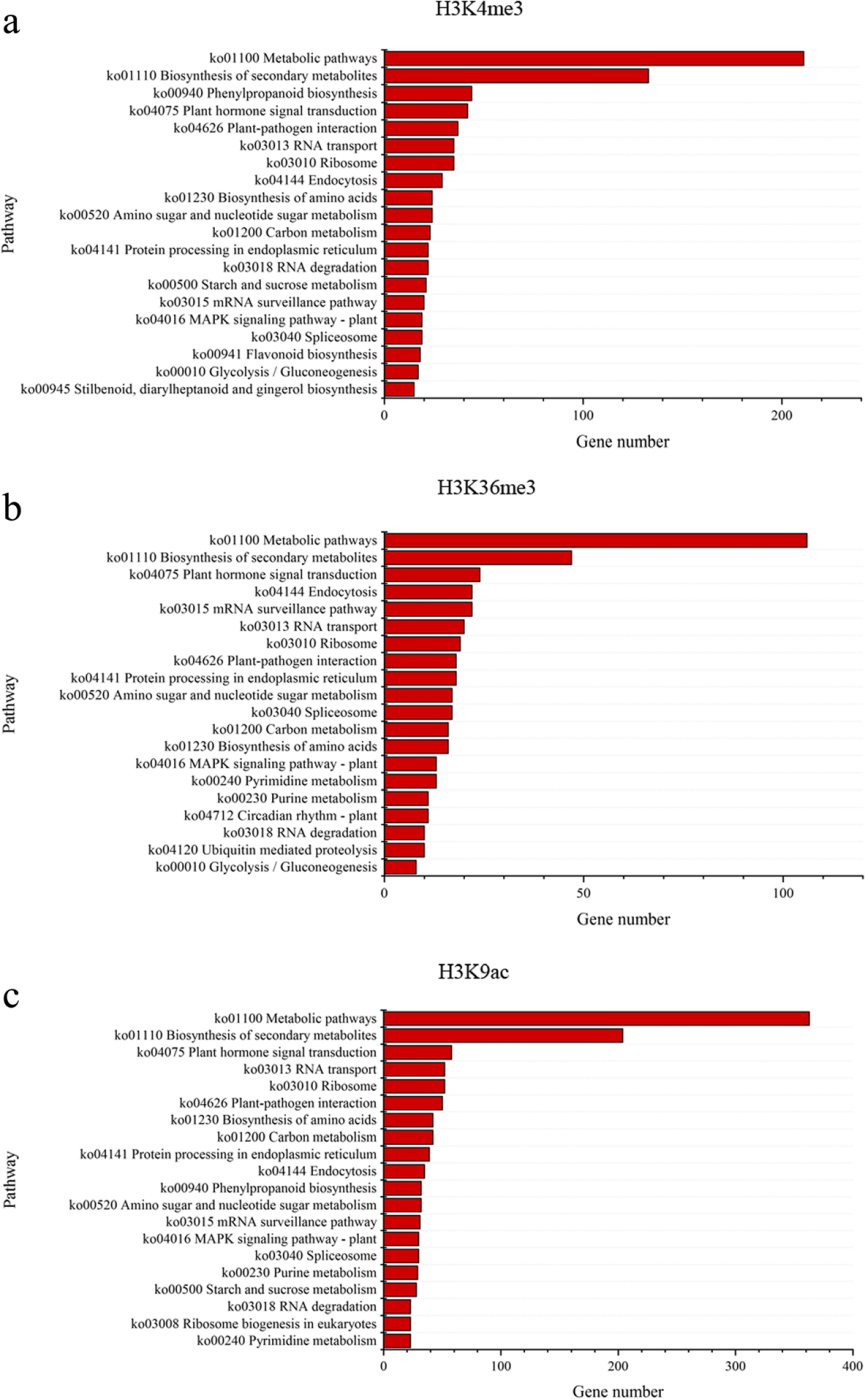
Pathway analysis of differentially modified genes by H3K4me3 (**a**), H3K36me3 (**b**) and H3K9ac (**c**) marks under phytoplasma stress. Top 20 pathways are shown in the figure

**Fig. 10:**
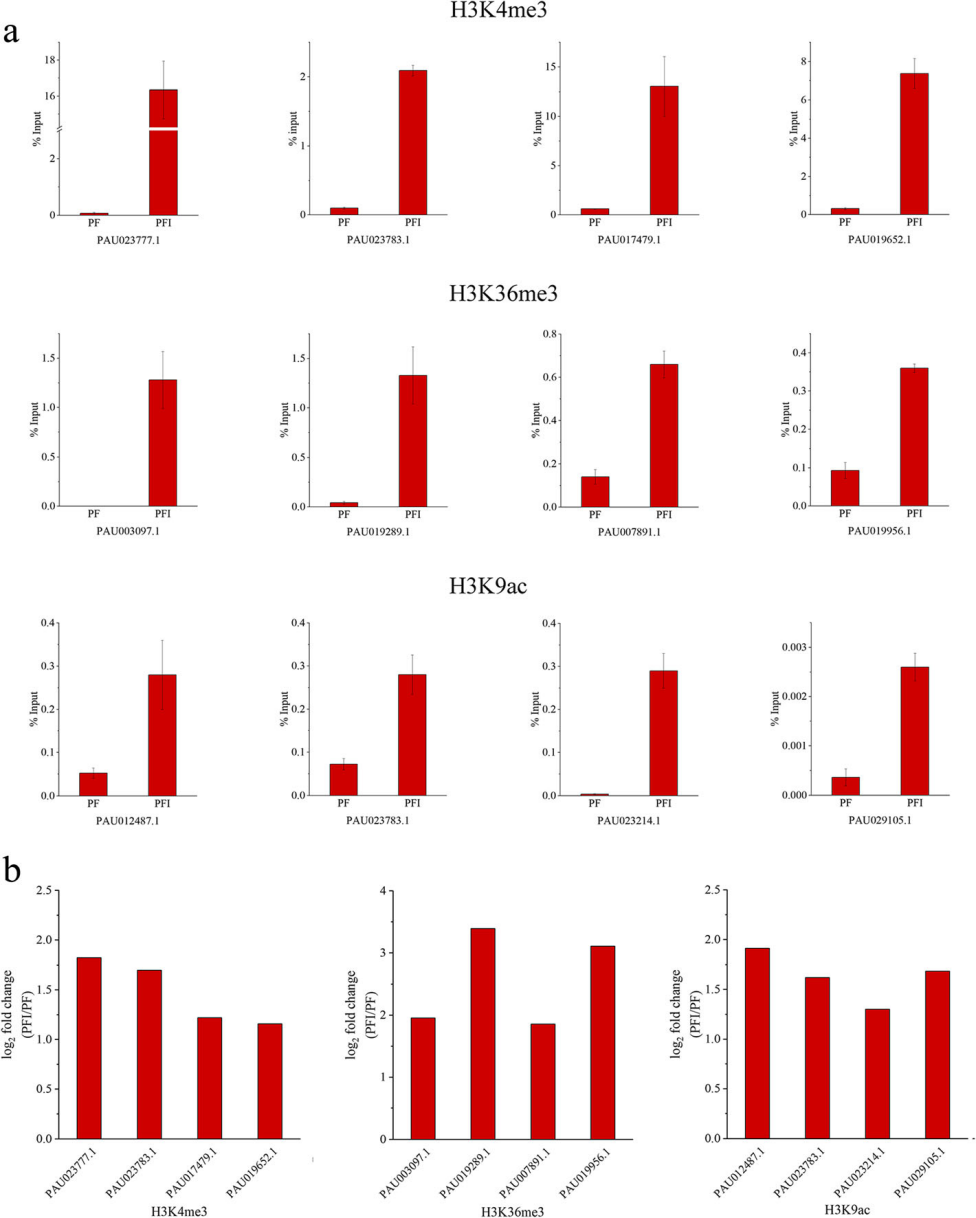
Validation of the ChIP-Seq analysis by ChIP-qPCR. **a** The results obtained by ChIP-qPCR. The DNA levels were normalized to input. H3K4me3: PAU023777.1, transcription factor MYC2; PAU023783.1, senescence-induced receptor-like serine/threonine-protein kinase; PAU017479.1, WRKY transcription factor 33; PAU019652.1, calcium-binding protein CML. H3K36me3: PAU003097.1, calmodulin; PAU019289.1, cyclic nucleotide gated channel; PAU007891.1, trans-cinnamate 4-monooxygenase; PAU019956.1, cinnamyl-alcohol dehydrogenase. H3K9ac: PAU012487.1, disease resistance protein RPM1; PAU023783.1, senescence-induced receptor-like serine/threonine-protein kinase; PAU023214.1, nitric-oxide synthase; PAU029105.1, trans-cinnamate 4-monooxygenase. **b** Changes of histone modifications derived from ChIP-Seq

In order to find out significantly changed histone modification locations in *P. fortunei* after phytoplasma infection, we compared ChIP read counts between PF and PFI for the H3K4me3, H3K9ac and H3K36me3 marks, respectively. The results showed that 1821, 1159, and 2727 regions were differentially marked by H3K4me3, H3K36me3, and H3K9ac in response to phytoplasma, respectively. The histone modification levels increased when Paulownia attacked by phytoplasma at most of these regions (Fig. 6; Additional file 2: Table S3). And 1738, 986, and 2577 genes were identified as differentially targeted by H3K4me3, H3K36me3, and H3K9ac, respectively. Interestingly, we observed that 141 differentially marked genes (DMGs) were co-modified by these three histone marks, and 393, 117 and 164 genes were co-occupied by at least two of these three histone marks, respectively. These results suggested that these histone marks may correlate together to regulate these genes (Fig. 7) [57, 62]. What’s more, DMGs co-modified by H3K4me3 and H3K9ac marks took a large proportion, indicating a cooperative interaction between these two histone marks, and this may be attributed to the similar distribution and function of these two histone marks on genes. GO analysis indicated that many of DMGs were involved in response to phytoplasma infection, such as participating in metabolic process, cellular process and response to stimulus (Fig. 8). KEGG analysis further showed that most of these genes were associated with metabolic pathways, biosynthesis of secondary metabolites, phenylpropanoid biosynthesis, plant-pathogen interaction and plant hormone signal transduction (Fig. 9). Furthermore, ChIP-qPCR was performed on three biological replicates of PF and PFI to confirm the results of ChIP-Seq. As was presented in Fig. 10, the ChIP-Seq results obtained in this study were validated to be acceptable by ChIP-qPCR.

### Correlation analysis of histone modification alterations with differential gene expression in response to phytoplasma in Paulownia

In our previous study, we have investigated the transcriptional changes in *P. fortunei* under phytoplasma stress, and significantly differentially expressed genes (DEGs) responsive to phytoplasma infection were identified by using the criteria of |log_2_ ratio| ≥ 1 and FDR < 0.001 [63]. Previous studies have proposed that H3K4me3, H3K9ac and H3K36me3 marks are associated with transcriptional activation. Here, we used these transcriptome datasets to examine whether the gene expression changes correlate with the changes of histone modifications in PFI. By using our RNA-Seq and ChIP-Seq datasets, we found that only 16.8% (292 of 1738), 18.1% (178 of 986), 16.8% (434 of 2577) genes differentially modified by H3K4me3, H3K36me3, and H3K9ac, were also responsive to phytoplasma at transcriptional level, respectively (data not shown). These results indicated that the transcriptional regulation of histone modifications only affected a small portion of phytoplasma-responsive genes. KEGG analysis suggested that these genes mainly participated in metabolic pathways, biosynthesis of secondary metabolites, phenylpropanoid biosynthesis, endocytosis, plant-pathogen interaction or plant hormone signal transduction (Additional file 3: Figure S1). Next, by comparing ChIP-Seq dataset with our previous DNA methylation and microRNA (miRNA) datasets, we noticed that 50.2% (872 of 1738), 41.5% (409 of 986) and 53.4% (1376 of 2577) genes differentially targeted by H3K4me3, H3K36me3 and H3K9ac respectively were also differentially modified by DNA methylation (data not shown), and 2.0% (34 of 1738), 1.2% (12 of 986) and 1.6% (40 of 2577) genes differentially occupied by H3K4me3, H3K36me3 and H3K9ac respectively were differentially targeted by miRNAs as well (data not shown). KEGG analysis revealed that these genes were mainly involved in metabolic pathways, biosynthesis of secondary metabolites, plant-pathogen interaction, RNA transport, phenylpropanoid biosynthesis or plant hormone signal transduction (Additional file 4: Figure S2; Additional file 5: Figure S3). Taken together, these results indicated a multi-level regulation of gene expression in response to phytoplasma infection. In addition to histone modifications, another two epigenetic regulation mechanisms, DNA methylation and small RNAs may also play an important role in gene expression.

To examine the influence of histone modifications on gene expression after phytoplasma infection, we focused on the DMGs in PFI with corresponding changes in gene expression. We noticed that 9 genes involved in plant-pathogen interaction, 7 genes participating in plant hormone signal transduction and 5 genes associated with phenylpropanoid biosynthesis showed alterations in both gene expression and histone modification (Additional file 6: Table S4; Additional file 7: Table S5; Additional file 8: Table S6), which may function in Paulownia defense response to phytoplasma, including brassinosteroid insensitive 1-associated receptor kinase 1 (BAK1, PAU001471.1), calcium-binding protein CML (CML, PAU019652.1), WRKY transcription factor 33 (WRKY 33, PAU017479.1), disease resistance protein RPM1 (PAU012487.1), transcription factor MYC2 (PAU023777.1), abscisic acid receptor PYR/PYL family (PAU019318.1), trans-cinnamate 4-monooxygenase (CYP73A, PAU022387.1, PAU029105.1, PAU007891.1), and shikimate O-hydroxycinnamoyltransferase (HCT, PAU019079.1).

## Discussion

PaWB disease is a serious infectious disease caused by phytoplasma, leading great economic loss for Paulownia industry. Our previous studies have provided valuable information on the molecular response of Paulownia to phytoplasma [49–52]. Recently, a growing body of evidence suggests that histone methylation and acetylation have all been implicated in the transcriptional regulation of plant defense responses against pathogens infection [20]. And thus, it is likely that the defense responses of Paulownia to phytoplasma would also be linked to the changes in the three well-studied histone marks, H3K4me3, H3K36me3 and H3K9ac, which positively correlate with gene expression. However, very limited information is available about this. To address this question, we investigated the genome-wide modification patterns of H3K4me3, H3K36me3 and H3K9ac in Paulownia, and compared them between PF and PFI by using ChIP-Seq approach. Meanwhile, the genome-wide modification changes of these histone marks were also compared with the genome-wide differential gene expression profiles.

### Genome-wide histone modification patterns in Paulownia

In the current study, we found that the three histone marks H3K4me3, H3K36me3 and H3K9ac were predominantly enriched in the genic regions in PF and PFI. However, the ratios of the three histone marks in each genomic region depended on modification type and altered after phytoplasma infection, which was highly in line with the histone marks distribution in other plants that already had epigenomic maps [58, 60, 61]. The concurrence frequencies analysis of the three histone modifications and gene expression indicated that a large proportion of genes in PF and PFI were co-modified by two or three histone marks. Moreover, high concurrence frequencies were observed between gene expression and the three histone modifications, suggesting that these histone marks may play positive roles as interrelated way in transcriptional regulation of Paulownia.

### Defense-related genes differentially marked by histone methylation and acetylation in response to phytoplasma in Paulownia

Plant defense response involves in a series of signaling cascades and is accomplished by a set of regulatory transcription factor cascades. It was reported that plant utilize Ca^2+^ signal as a vital early signaling event in response to pathogen recognition. After pathogen perception, free Ca^2+^ concentrations increases in the cytosol of plant governed by cyclic nucleotide-gated ion channels (CNGCs), which is a pivotal event in the activation of plant defense responses, facilitating the induction of ROS and activation of mitogen-activated protein kinase (MAPK) cascades [64]. CaM-like proteins (CMLs) are a class of Ca^2+^-binding sensor relays, transducing the Ca^2+^ signal to downstream targets during this signal transduction cascade, through the Ca^2+^-induced conformational changes and then interacting with their target proteins [65]. Accumulating evidence shows that these Ca^2+^ sensors were involved in signaling cascade of the plant responses to pathogens. It was reported that the immune responses of plants would be strongly affects in the mutated plants whose CaM/CML gene expression was deregulated or CaM/CML function was lost. For instance, Takabatake et al. [66] reported that an enhanced susceptibility of tobacco plants to tobacco mosaic virus (TMV), bacterial pathogen *Ralstonia solanacearum* and fungal pathogens *Rhizoctonia solani* and *Pythium aphanidermatum* was observed when *NtCaM13*, a gene encoding CaM, was silenced. Chiasson et al. [67] found that reducing expression of a CML encoding gene (*APR134*) in tomato resulted in the impairment of HR, while transgenic Arabidopsis overexpressing *AtCML43* (an orthologous gene of *APR134* in Arabidopsis) accelerated the HR in response to a *Pseudomonas syringae* pv. *tomato*. Leba et al. [68] demonstrated that *Arabidopsis thaliana* CML9 participates in plant defense responses and may fine-tune plant defense processes with the evidence that the expression of *CML9* was rapidly induced in response to *Pseudomonas syringae*, and the responses to pathogen observed normally in the wild-type Arabidopsis were altered in *CML9*-knockout mutants and transgenic lines over-expressing *CML9*. Here, we found that the expression of gene encoding calcium-binding protein CML (PAU019652.1) was induced in response to phytoplasma, and it was also differentially occupied by H3K4me3, and H3K9ac marks, suggesting that these two histone marks may regulate the calcium signaling in Paulownia under phytoplasma stress.

WRKY proteins, which are characterized by the presence of the highly conserved WRKY domain, are a large superfamily of transcription factors (TFs) in plants with pivotal roles in the regulation of transcriptional reprogramming associated with plant immune responses to pathogen infection. WRKY transcription factor 33 (WRKY33) belonging to group I WRKY protein family is a member of WRKY TFs that have been reported to participate in the immune response to pathogens. Zheng et al. [69] reported that the expression of *WRKY33* was induced after pathogen infection, and over-expression of *WRKY33* in transgenic plants or loss of *WRKY33* function in mutated plants altered Arabidopsis responses to *Pseudomonas syringae* and necrotrophic pathogens. For example, increased resistance to *Botrytis cinerea* and *Alternaria brassicicola* was observed in *WRKY33*-overexpressing Arabidopsis, while *wrky33* mutants of Arabidopsis showed enhanced susceptibility to these two pathogens. And similar phenomena were also observed in grapevine defense against the oomycete pathogen *Plasmopara viticola* [70] and oilseed rape resistance to *Sclerotinia sclerotiorum* [71]. Additionally, recent evidence suggested that *AtWRKY33* was required for the induction of PHYTOALEXIN DEFICIENT3 (PAD3) and the production of pathogen-induced antimicrobial camalexin, which was a major phytoalexin in Arabidopsis, depending on the activation of MPK3/MPK6 cascade [72], and it directly targeted genes associated with redox homeostasis, SA signaling, ethylene-JA-mediated cross-communication, and camalexin biosynthesis [73]. In our transcriptome data, a WRKY33 encoding gene (PAU017479.1) was found to be significantly induced by phytoplasma, and its H3K4me3 level was correspondingly increased in response to phytoplasma.

Previous studies showed that the biosynthesis of lignin could be induced under various stress conditions, including pathogen invasion, except for developmentally programmed deposition, in that lignification and reinforcement of cell walls are important processes in the responses of plants to pathogen infection [74–76]. Here, we found that three genes encoding CYP73A (PAU022387.1, PAU029105.1, PAU007891.1) and a gene encoding HCT (PAU019079.1) were significantly induced by phytoplasma, and their histone modification levels (H3K4me3, H3K9ac or H3K36me3) altered in response to phytoplasma. Hence, we speculated that the lignin biosynthesis of *P. fournei* was activated after phytoplasma infection, which might enhance its capacity for lignification to restrict pathogen invasion, and histone modifications may exert their regulatory roles in the expression of these lignin biosynthesis-related genes in this process.

### The potential roles of histone modifications on gene expression

H3K4me3, H3K36me3 and H3K9ac are three well-characterized histone marks which have been demonstrated to be associated with gene activation. In our study, histone modifications only affected the expression of a small portion of phytoplasma-responsive genes. Among these genes, only a small number of them displayed alterations in histone modification paralleled by the changes in gene expression [63] in *P. fortunei* infected with phytoplasma. And some DEGs were found to be co-occupied by more than one histone marks, indicating the possible relevance of the balance of different histone marks to the transcriptional regulation in Paulownia-phytoplasma interaction. However, we noticed that changes of histone modification for some genes in phytoplasma-infected *P. fortunei* disaccorded to the changes in gene expression. This phenomenon can be attributed to the complex regulation of gene expression [77–79]. In addition to histone modifications, DNA methylation and small RNAs also can have effects on gene expression. Compared the histone modifications data in our ChIP-Seq with our previous DNA methylation data obtained by whole genome bisulfite sequencing (WGBS-Seq) (unpublished), we found that the majority of genes differentially modified with H3K4me3 (872), H3K36me3 (409), and H3K9ac (1376) also contained one or more differential DNA methylation regions. Small RNAs are considered to be the gene repressors in the transcriptional or posttranscriptional way [80]. In other words, the downregulation and upregulation of small RNAs in phytoplasma-infected *P. fortunei* could result in the upregulation and downregulation of their target genes. These observations indicated that DNA methylation and small RNAs add the other two layers of regulatory machinery to the complex gene expression regulation network of Paulownia in response to phytoplasma.

## Conclusions

In summary, our genome-wide investigation of three major histone marks (H3K4me3, H3K36me3 or H3K9ac) showed that these histone marks appeared mainly in genic regions of PF and PFI. Many defense-related genes were differentially modified with H3K4me3, H3K36me3 or H3K9ac after phytoplasma infection. A small number of these genes was in concert with changes in histone modification and gene expression, including CML, WRKY 33, disease resistance protein RPM1, MYC2, abscisic acid receptor PYR/PYL family, CYP73A, HCT. These results suggested that histone modifications may play significant but largely unknown roles in the defense response of *P. fortunei* against phytoplasma, and the underlying regulatory mechanism needs to be further investigated in the future.

## Methods

### Plant materials

*P. fortunei* with (PFI) and without (PF) Paulownia witches’ broom phytoplasma (Aster Yellows group, 16SrI-D) infection were used in this study. The tissue-cultured plantlets with 30-day-old for each sample were used in this study as previously described in the method of Fan et al. [81] and Yao et al. [82]. The tissue-culture condition as follow: 25 ± 2 °C, relative humidity of 70%, illumination intensity of 130 μmol·m^− 2^·s^− 1^ and photoperiod of 16-h-light/8-h-dark. The terminal buds of PF and PFI plantlets were harvested. Three independent biological replicates were prepared for ChIP-Seq library construction.

### Chromatin immunoprecipitation

The chromatin immunoprecipitation (ChIP) were performed based on the protocol as previously described by Zong et al. [83] with minor modifications. Briefly, terminal buds from PF and PFI plantlets were firstly cross-linked with 1% formaldehyde in a vacuum for 15 min. This step was terminated by adding glycine to a final concentration of 0.125 M and another vacuum was applied for 5 min. After rinsing and drying, the terminal buds of each sample were finely ground to a powder in liquid nitrogen. Subsequently, the chromatin was isolated, sonicated, and immunoprecipitated against anti-H3K4me3 antibody (abcam), anti-H3K36me3 antibody (abcam) and anti-H3K9ac antibody (abcam), respectively. The immune complex was washed, eluted and reversely cross-linked. The immunoprecipitated DNA was recovered in the method of phenol-chloroform extraction, then purified and dissolved in distilled water. Corresponding sample handled without addition of any antibody was severed as input control. The ChIP DNA and input DNA were used to construct ChIP-seq libraries using NEXTflex® Rapid DNA-Seq Kit (Bioo Scientific, Austin, TX, USA) following the manufacturer’s procedure. The libraries were sequenced on Illumina Hiseq 4000 platform for 150 bp paired-end sequencing. These processes were conducted by Wuhan Igenebook Biotechnology Co., Ltd. (www.igenebook.com). Three independent biological replicates of ChIP-Seq for each sample were performed.

### ChIP-Seq data analysis

The raw reads were first cleaned up to obtain high-quality clean reads. The clean reads were mapped to the *P. fortunei* reference genome (http://paulownia.genomics.cn/page/species/index.jsp) using BWA software with default parameters [84].Reproducibility between biological replicates was assessed using the Pearson correlation for the genome-wide reads distribution at each pair of replicates. Genomic regions associated with histone modification were identified using Model-based Analysis of ChIP-Seq (MACS) by default parameters (bandwidth, 300 bp; model fold, 10, 30; *p* value, 1.00e-5) [85]. The absolute value of log_2_ ratio ≥ 1 and *p*-value < 0.01 was used as a criterion to identify significant differential histone modified regions. GO and KEGG functional analysis of modified genes were performed as described previously [86].

### ChIP-qPCR validation

To validate the ChIP-Seq results, we randomly selected four genes differentially marked by H3K4me3, H3K36me3 and H3K9ac from ChIP-Seq analysis for ChIP-qPCR validation. The PCR primers of the genes were designed using Primer premier 5.0 software (Premier Biosoft International, Palo Alto, CA) and presented in Additional file 9: Table S2. The ChIP-qPCR was performed on Applied Biosystems 7300 real-time PCR System (Applied Biosystems, Foster City, CA). The ChIP-qPCR was performed in 20 μl reactions using ChamQ SYBR Color qPCR Master Mix (Vazyme Biotech Co., Ltd., Nanjing, Jiangsu, China). The reactions consisted of 10 μl 2× ChamQ SYBR Color qPCR Master Mix, 3 μl DNA template, 1 μl forward and reverse primer, and 5 μl ddH_2_O. The amplification procedure was as follows: 95 °C for 30 s, 40 cycles of 95 °C for 10 s and 60 °C for 30 s, followed by 95 °C for 15 s, 60 °C for 60s and 95 °C for 15 s. ChIP DNA enrichment was determined as % of input in the method of Mukhopadhyay et al. [87]. Three biological replicates of each sample were assayed for ChIP-qPCR.

## Additional files

Additional file 1: **Table S1.** Summary statistics of ChIP-Seq for H3K4me3, H3K36me3 and H3K9ac in Paulownia (DOCX 14 kb)

Additional file 2: **Table S3.** Differentially marked histone methylation and acetylation regions between *P. fortunei* and *P. fortunei* with phytoplasma infection (XLSX 1028 kb)

Additional file 3: **Figure S1.** Pathway analysis of differentially (a) H3K4me3-, (b) H3K36me3- and (c) H3K9ac-modified genes with altered expression at transcriptional level under phytoplasma stress. Top 20 pathways are shown in the figure. (TIF 1528 kb)

Additional file 4: **Figure S2.** Pathway analysis of genes differentially modified by both DNA methylation and H3K4me3 (a), H3K36me3 (b) and H3K9ac (c) marks under phytoplasma stress. Top 20 pathways are shown in the figure. (TIF 1511 kb)

Additional file 5: **Figure S3.** Pathway analysis of genes differentially targeted by both miRNAs and H3K4me3 (a), H3K36me3 (b) and H3K9ac (c) marks under phytoplasma stress (TIF 1082 kb)

Additional file 6: **Table S4.** List of genes involved in plant-pathogen interaction from ChIP-Seq analysis (XLSX 24 kb)

Additional file 7: **Table S5.** List of genes involved in plant hormone signal transduction from ChIP-Seq analysis (XLSX 28 kb)

Additional file 8: **Table S6.** List of genes involved in phenylpropanoid biosynthesis from ChIP-Seq analysis (XLSX 20 kb)

Additional file 9: **Table S2.** Primer sequences of genes used for ChIP-qPCR (DOCX 14 kb)

## Abbreviations

BAK1: Brassinosteroid insensitive 1-associated receptor kinase 1
ChIP-Seq: Chromatin immunoprecipitation sequencing
CML: Calcium-binding protein CML
CNGCs: Cyclic nucleotide-gated ion channels
CYP73A: Trans-cinnamate 4-monooxygenase
DEGs: Differentially expressed genes
DMGs: Differentially marked genes
ETI: Effector-triggered immunity
HCT: Shikimate O-hydroxycinnamoyltransferase
HDACs: Histone deacetylases
HR: Hypersensitive response
lncRNAs: Long noncoding RNAs
LRR-RLKs: Leucine-rich repeat transmembrane receptor-like kinases
MAPK: Mitogen-activated protein kinase
miRNAs: Micro RNAs
PaWB: Paulownia withes’-broom
PF: *P. fortunei* without phytoplasma infection
PFI: *Paulownia fortunei* with phytoplasma infection
PR1: Pathogenesis related 1
PRRs: Pattern recognition receptors
PTI: Pathogen-associated molecular pattern-triggered immunity
ROS: Reactive oxygen species
SDG8: SET domain group 8
WRKY 33: WRKY transcription factor 33
