# Supplementary material for: Genome-wide analysis of three histone marks and gene expression in Paulownia fortunei with phytoplasma infection
Source: BMC Genomics. 2019 Mar 21;20:234. doi: 10.1186/s12864-019-5609-1 (PMC6429711; doi:10.1186/s12864-019-5609-1)
Supplement: Supplementary file 1 — Table S1. Summary statistics of ChIP-Seq for H3K4me3, H3K36me3 and H3K9ac in Paulownia (DOCX 14 kb) [file 12864_2019_5609_MOESM1_ESM.docx]

**Table S1 Summary statistics of ChIP-Seq for H3K4me3, H3K36me3 and H3K9ac in Paulownia**

|  |  | **H3K4me3** | | **H3K36me3** | | **H3K9ac** | |
| --- | --- | --- | --- | --- | --- | --- | --- |
|  | **Sample** | **PF** | **PFI** | **PF** | **PFI** | **PF** | **PFI** |
| **Raw reads** | **Rep1** | 54,123,668 | 60,934,130 | 42,017,810 | 56,643,522 | 56,115,430 | 41,109,982 |
|  | **Rep2** | 41,397,162 | 50,843,928 | 42,439,836 | 49,956,598 | 42,153,150 | 55,329,522 |
|  | **Rep3** | 45,741,332 | 42,257,778 | 59,719,266 | 53,573,012 | 67,750,764 | 50,525,850 |
| **Clean reads** | **Rep1** | 48,475,104 | 49,365,138 | 38,366,562 | 48,090,456 | 49,807,020 | 34,507,242 |
|  | **Rep2** | 37,125,072 | 43,441,644 | 38,853,218 | 43,361,398 | 38,319,132 | 46,957,708 |
|  | **Rep3** | 42,136,418 | 38,563,034 | 54,852,616 | 48,276,648 | 62,142,222 | 45,052,572 |
| **Mapped reads** | **Rep1** | 43,364,853  (89.46%) | 41,775,227  (84.62%) | 34,660,769  (90.34%) | 41,680,645  (86.67%) | 43,174,094  (86.68%) | 27,512,143  (79.73%) |
|  | **Rep2** | 32,454,638  (87.42%) | 36,636,987  (84.34%) | 34,884,801  (89.79%) | 37,257,911  (85.92%) | 33,791,935  (88.19%) | 37,700,863  (80.29%) |
|  | **Rep3** | 38,287,959  (90.87%) | 34,253,475  (88.82%) | 49,370,111  (90.01%) | 42,236,832  (87.49%) | 53,774,517  (86.53%) | 34,431,296  (76.42%) |
