# Supplementary figures and images for: Genome-wide analysis of three histone marks and gene expression in Paulownia fortunei with phytoplasma infection
Source: BMC Genomics. 2019 Mar 21;20:234. doi: 10.1186/s12864-019-5609-1 (PMC6429711; doi:10.1186/s12864-019-5609-1)

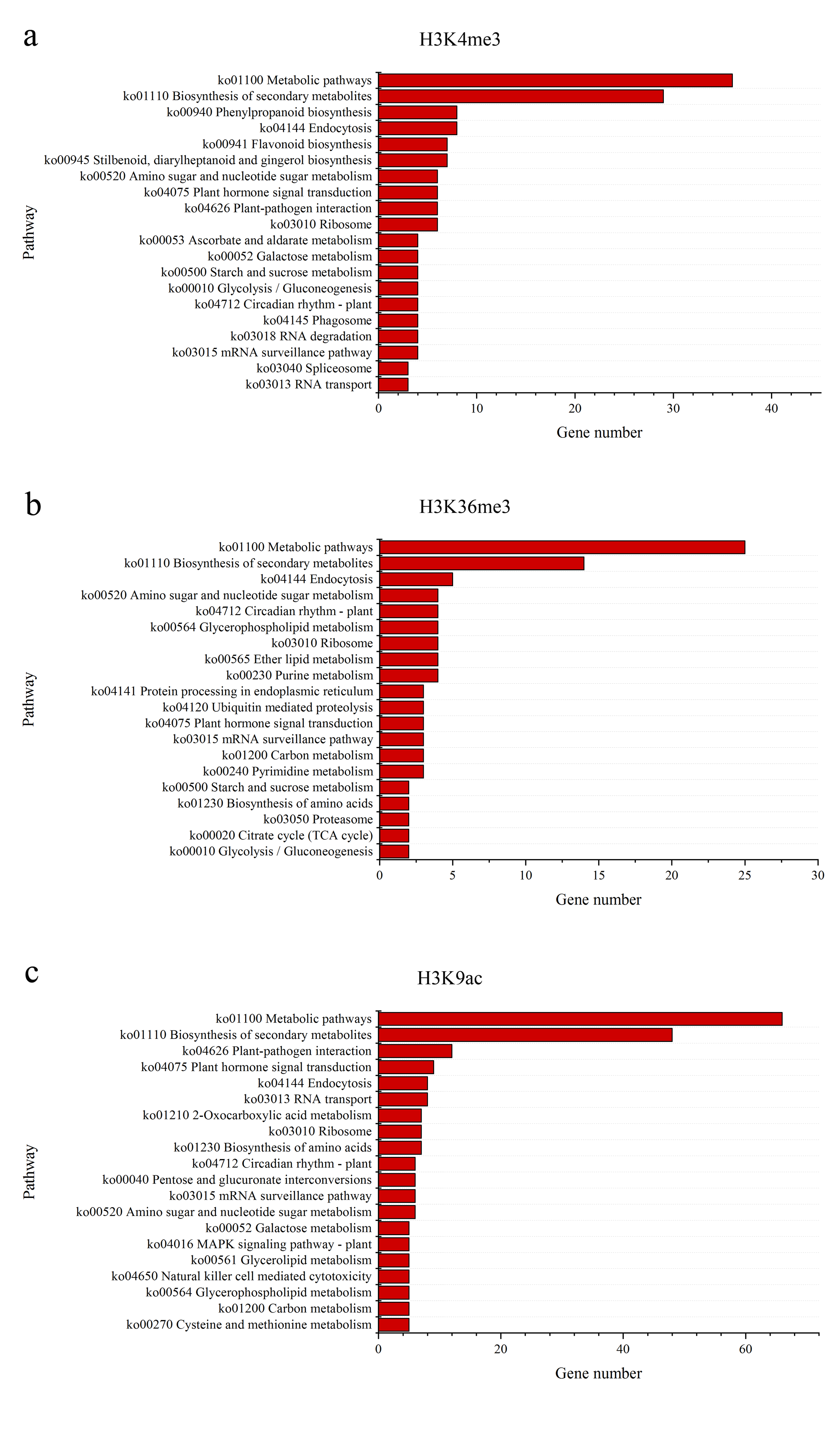

Supplement: Supplementary file 3 — Figure S1. Pathway analysis of differentially (a) H3K4me3-, (b) H3K36me3- and (c) H3K9ac-modified genes with altered expression at transcriptional level under phytoplasma stress. Top 20 pathways are shown in the figure. (TIF 1528 kb) [file 12864_2019_5609_MOESM3_ESM.tif]

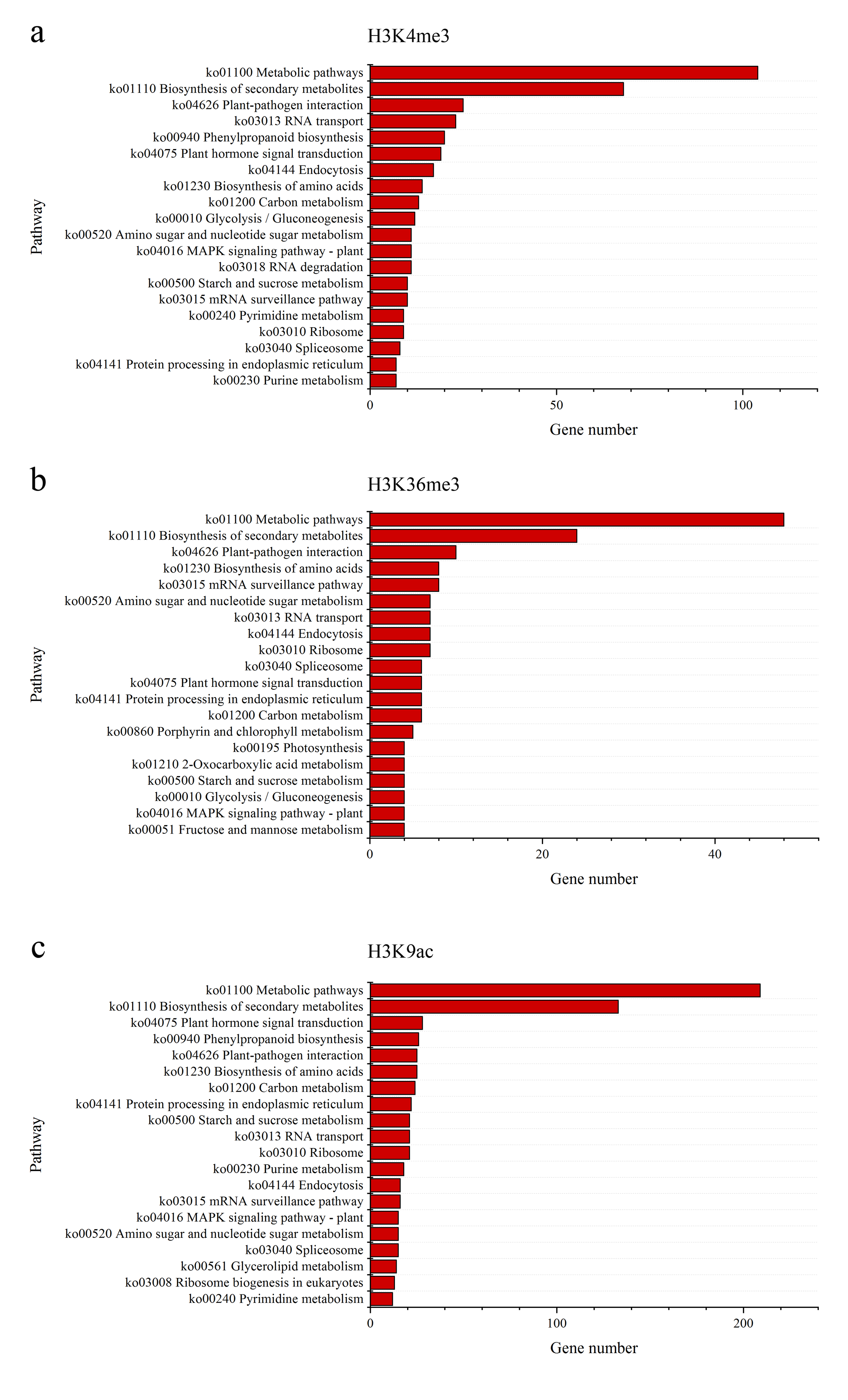

Supplement: Supplementary file 4 — Figure S2. Pathway analysis of genes differentially modified by both DNA methylation and H3K4me3 (a), H3K36me3 (b) and H3K9ac (c) marks under phytoplasma stress. Top 20 pathways are shown in the figure. (TIF 1511 kb) [file 12864_2019_5609_MOESM4_ESM.tif]

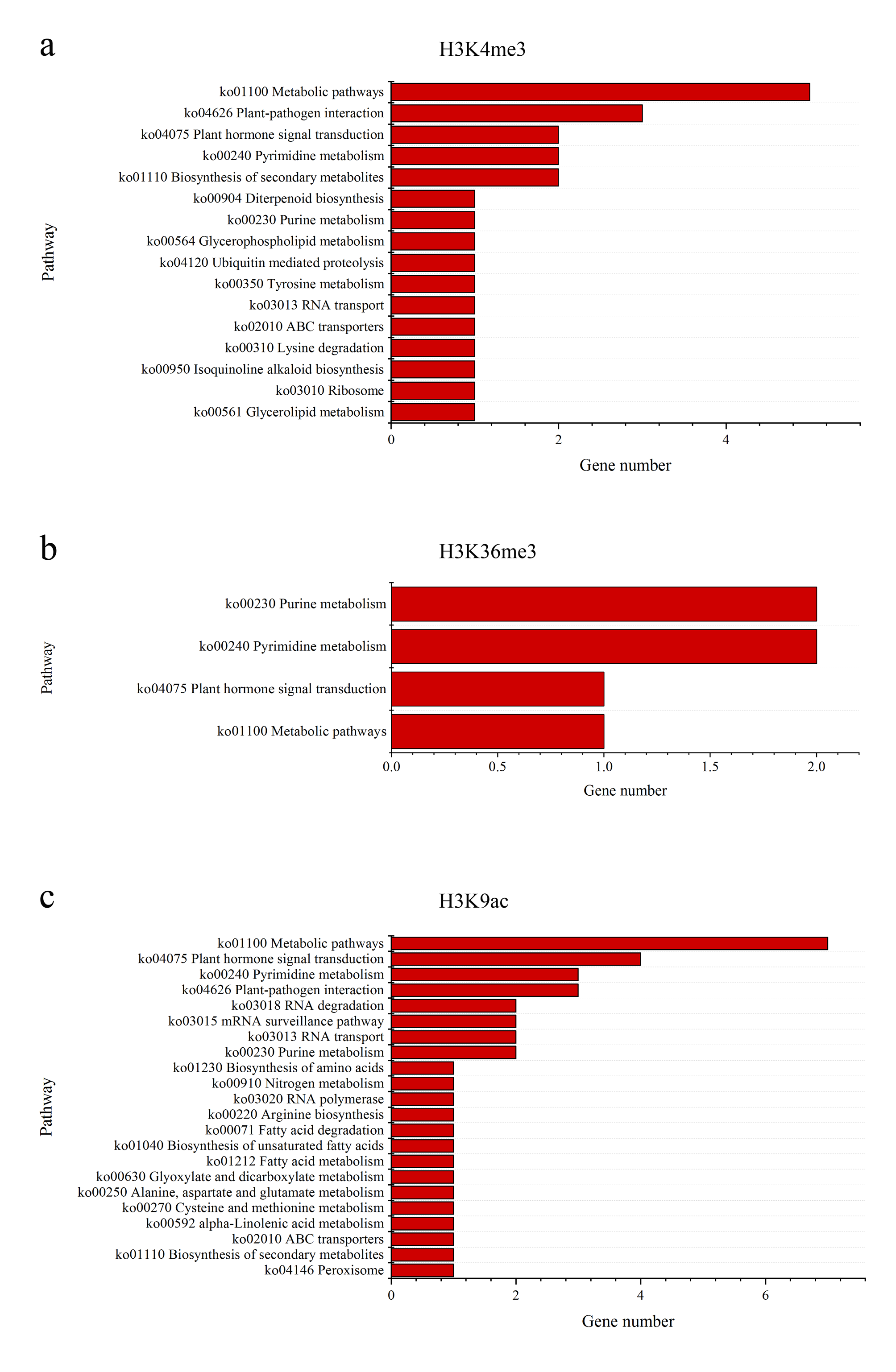

Supplement: Supplementary file 5 — Figure S3. Pathway analysis of genes differentially targeted by both miRNAs and H3K4me3 (a), H3K36me3 (b) and H3K9ac (c) marks under phytoplasma stress (TIF 1082 kb) [file 12864_2019_5609_MOESM5_ESM.tif]
