# Supplementary material for: Genome-wide analysis of three histone marks and gene expression in Paulownia fortunei with phytoplasma infection
Source: BMC Genomics. 2019 Mar 21;20:234. doi: 10.1186/s12864-019-5609-1 (PMC6429711; doi:10.1186/s12864-019-5609-1)
Supplement: Supplementary file 9 — Table S2. Primer sequences of genes used for ChIP-qPCR (DOCX 14 kb) [file 12864_2019_5609_MOESM9_ESM.docx]

**Table S2 Primer sequences of genes used for ChIP-qPCR**

| **Marks** | **Gene ID** | **Primer sequences** | |
| --- | --- | --- | --- |
| H3K4me3 | PAU023777.1 | TTCGGATACGATGGCACG | CACTGTGGCTTTTGGAGGCT |
|  | PAU023783.1 | GTCGTCGTCATTCCGTTTAC | TGGTAAGTGGTGGTGCTGTC |
|  | PAU017479.1 | TCTGCTGAAACCCAAACTCC | GAAAATGACCCGGACAATGA |
|  | PAU019652.1 | CAGGATTTCTTGCGGTTGAT | CATCATACGATTTCGGGTCC |
| H3K36me3 | PAU003097.1 | AATGGCAGAAGAAGAGGGTC | GAAAAGGAAAAAAGCCAAGA |
|  | PAU019289.1 | GTTTAGAGGGAGGGGAGGC | GGTTGGAGGTGGGGGGT |
|  | PAU007891.1 | GTGGTTCAAGTCATCGCCTAC | ATCGTCGCCACCGTTGT |
|  | PAU019956.1 | AAGTGTCTCAACGGGCTGTA | GCGGACGAGGATTTCATT |
| H3K9ac | PAU012487.1 | TCAGGGTTTGGGTGAAGC | CCTGTGGCTTTGATGATTCT |
|  | PAU023783.1 | GTCGTCGTCATTCCGTTTA | TGGTAAGTGGTGGTGCTGT |
|  | PAU023214.1 | TGGAAACAAGGGCAGAAC | AAATGGATGAAGGGTCGA |
|  | PAU029105.1 | CATCCAACCCACAAGTCGG | ACCCAGCATTCCATCCAAAC |
